# Survey of the infant male urobiome and genomic analysis of *Actinotignum* spp.

**DOI:** 10.1038/s41522-023-00457-6

**Published:** 2023-12-01

**Authors:** Seth A. Reasoner, Viktor Flores, Gerald Van Horn, Grace Morales, Leslie M. Peard, Benjamin Abelson, Carmila Manuel, Jessica Lee, Bailey Baker, Timothy Williams, Jonathan E. Schmitz, Douglass B. Clayton, Maria Hadjifrangiskou

**Affiliations:** 1 Division of Molecular Pathogenesis, Department of Pathology, Microbiology & Immunology, Vanderbilt University Medical Center, Nashville, TN USA; 2 Division of Pediatric Urology, Vanderbilt University Medical Center, Nashville, TN USA; 3 Center for Personalized Microbiology (CPMi), Vanderbilt University Medical Center, Nashville, TN USA; 4 Vanderbilt Institute for Infection, Immunology and Inflammation, Vanderbilt University Medical Center, Nashville, TN USA; 5 Department of Urology, Vanderbilt University Medical Center, Nashville, TN USA; 6 Present Address: Division of Pediatric Urology, Phoenix Children’s Hospital, Phoenix, AZ USA

**Keywords:** Microbiome, Next-generation sequencing

## Abstract

The urinary bladder harbors a community of microbes termed the urobiome, which remains understudied. In this study, we present the urobiome of healthy infant males from samples collected by transurethral catheterization. Using a combination of enhanced culture and amplicon sequencing, we identify several common bacterial genera that can be further investigated for their effects on urinary health across the lifespan. Many genera were shared between all samples suggesting a consistent urobiome composition among this cohort. We note that, for this cohort, early life exposures including mode of birth (vaginal vs. Cesarean section), or prior antibiotic exposure did not influence urobiome composition. In addition, we report the isolation of culturable bacteria from the bladders of these infant males, including *Actinotignum* spp., a bacterial genus that has been associated with urinary tract infections in older male adults. Herein, we isolate and sequence 9 distinct strains of *Actinotignum* spp. enhancing the genomic knowledge surrounding this genus and opening avenues for delineating the microbiology of this urobiome constituent. Furthermore, we present a framework for using the combination of culture-dependent and sequencing methodologies for uncovering mechanisms in the urobiome.

## Introduction

Until the past decade, it was presumed that the healthy urinary bladder was a sterile environment. However, advances in genetic sequencing and enhanced culture methodologies have uncovered a resident microbiota of the bladder, and this community has been termed the urobiome^1–3^. Since the discovery of the urobiome, several studies have demonstrated connections between urobiome dysbiosis and a variety of genitourinary diseases, including nephrolithiasis^4–7^, recurrent UTIs^8^, female urinary incontinence^9–13^, interstitial cystitis^14–17^, overactive bladder^18,19^, male lower urinary tract symptoms^20^, bladder cancer^21,22^, and prostate cancer^23,24^. Likewise, efforts have been made to understand the healthy composition of the urobiome across the lifespan^25–27^. Despite a decade of research, little progress has been made to understand the development of the urobiome and its mechanistic interactions with urinary pathogens.

To begin to address the question of urobiome development, several studies have investigated the urobiome of children^28–34^. However, these studies sampled pediatric subjects with a variety of pre-existing urinary tract diseases or infections, which was the indication for urinary catheterization. For a variety of other anatomic niches, including the skin and gastrointestinal tract, early life development of the resident microbiota shapes future microbial diversity and susceptibility to disease^35^. Therefore, defining the development of a healthy pediatric urobiome is a vital endeavor. To bridge this gap in the field and investigate the urinary microbiome of healthy infants, we collected catheterized urine samples under sterile operating room conditions at the time of circumcision of male infants under one year of age. Notably, none of the subjects had structural or functional urinary tract abnormalities or prior urinary tract infections. Thus, our study represents the first investigation of the healthy infant urobiome, albeit limited to the male gender. We provide evidence of a detectable and culturable urobiome of healthy infant males. Using complementary approaches of enhanced culture and 16S rRNA amplicon sequencing, we report a diverse and consistent urobiome signature in infant males that does not appear to be perturbed by early life exposures, such as mode of delivery (vaginal vs. Cesarean section) or prior antibiotic exposure for non-urinary infections. Among the urobiome residents cultured, we report *Actinotignum* as a genus of interest, because of its prevalence in both the adult and pediatric urobiomes^2,28,36–40^ and its implication as a uropathogen in certain patient populations^41–43^. To facilitate future mechanistic work on this urobiome member, we provide whole genome sequencing information of nine independent strains of *Actinotignum* spp.

Research investigations of the urobiome are still within their first decade. As ongoing research continues to define the urinary microbiome, standardized methods and reporting are vital^44^. Given the low biomass of the urinary microbiome, the potential for contamination during sample collection, processing, and analysis is high. In this work, we utilize both culture-dependent and independent methodologies to assess the infant urobiome. We present rigorous sampling and processing controls to benchmark the potential contaminants introduced during sample collection and processing. We include extensive methodological, bioinformatic, and statistical documentation to promote accessibility and reproducibility within the nascent urobiome field.

## Results

**Fig. 1:**
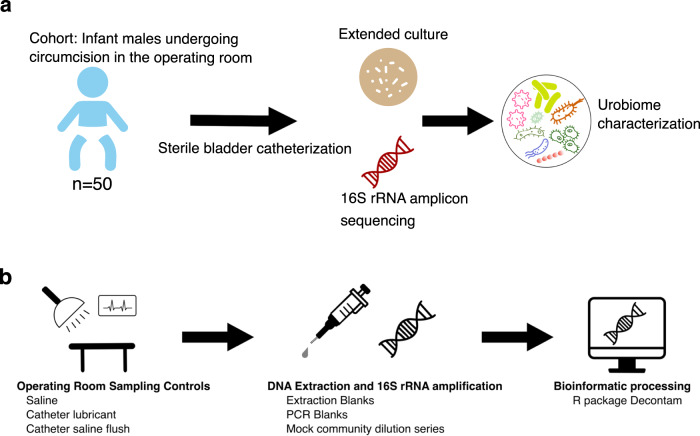
Study schematic and analysis workflow. **a** Illustration of study design. Fifty male infants were sterilely catheterized in the operating theater prior to undergoing circumcision. Urine was immediately plated for enhanced urine culture. DNA was extracted from urine samples, amplified with V4 16S rRNA primers, and sequenced using Illumina paired-end chemistry. The combination of urine culture and sequencing results was used to describe the urobiome composition. **b** Illustration of analysis workflow and evaluation of potential contaminant sources. Sampling controls were collected contemporaneously with urine samples in the operating theater. Extraction blanks and a mock microbial community dilution series were used to benchmark DNA extraction. No template blanks were subjected to 16S rRNA PCR amplification to benchmark PCR amplification. All controls mentioned were subjected to Illumina paired-end sequencing. The Decontam package in R was used to filter potential contaminant sequences.

This study aimed to bridge a gap in our knowledge of the healthy pediatric urobiome. Our study prospectively enrolled 50 healthy male infants who underwent urinary catheterization during routine operative circumcision (Fig. 1a). Below, we describe the pipeline we established to increase rigor and reproducibility within urobiome research, followed by a description of our findings.

### Establishing methodology for low biomass urine samples from infants

The method of sample collection is a key concern in urobiome research. Genital and intestinal contaminants confound urobiome results^44^. To obtain sterile catheterized samples from healthy infants, we selected the population of infant males undergoing circumcision in the operating room. Informed consent for bladder catheterization was obtained from parental guardians. We collected urine from 50 male infants following induction of general anesthesia and sterilization of the periurethral area. The median age of the infants was 215 days (~7 months old, Table 1). The average amount of urine collected was 5.81 mL (range 0.4–28 mL). Urine was immediately plated for enhanced urine culture as described in the methods to isolate and identify culturable bacteria. For sequencing, aliquots of the same urine were immediately frozen at −80 °C to prevent microbial growth or contamination prior to processing for sequencing.

**Table 1:** Cohort details.

Participants (*n*)	50
Median age (days) at sample collection (IQR)	215 (190, 252)
Male sex	100%
*Birth history*	
Cesarean section	27 (54%)
Preterm	18 (36%)
NICU following birth	18 (36%)
*Health exposures*	
Prior antibiotic exposure	12 (24%)
*Type of nutrition*	
Breast milk only	8 (16%)
Formula	22 (44%)
Breast milk and formula	18 (36%)
Solids or puree	14 (28%)
*Urine collection*	
Volume of urine (mean, range)	5.81 mL (0.4–28 mL)

We utilized two non-selective agar media (blood agar and Brucella agar) plated in duplicate and incubated under either an ambient atmosphere with 5% CO_2_ supplementation or anaerobic conditions. These media were chosen to broadly capture culturable members of the urobiome even fastidious members. Aliquots of the same urine were subjected to DNA extraction using a commercially available kit that utilizes bead beating and DNA binding by magnetic beads for DNA isolation and purification. Isolated DNA was amplified using standardized PCR primers for the V4 region of the 16S rRNA. 16S rRNA amplicons were sequenced by Illumina paired-end sequencing.

The urobiome is a low biomass environment. There are myriad potential sources of contamination, a concern that is accentuated for low biomass samples. Contamination can be introduced at any step of sample processing, from sample collection to DNA extraction and amplification to sequencing^45–47^. We utilized three types of negative controls: (1) DNA extraction blanks; (2) no template PCR amplification blanks; and (3) sampling controls (Fig. 1b). Specifically, we included eight DNA extraction blanks which underwent all steps of DNA extraction, PCR amplification, and sequencing. We included four no-DNA template blanks during PCR amplification of the V4 region of the 16S rRNA. Finally, we included three types of sampling controls (operating theater saline, mineral oil used for catheter lubrication, and saline flushed through a sterile catheter); four sets of which were collected on separate days.

We sequenced sampling controls on an Illumina NovaSeq 6000 to add additional resolution to rare contaminant sequences due to sequencing equipment availability. Urine samples were sequenced on an Illumina MiSeq. Notably, all samples and controls were processed in the same laboratory using the same reagents. To account for the different read numbers between MiSeq and NovaSeq platforms, we utilized a dilution series of a mock microbial community.

**Fig. 2:**
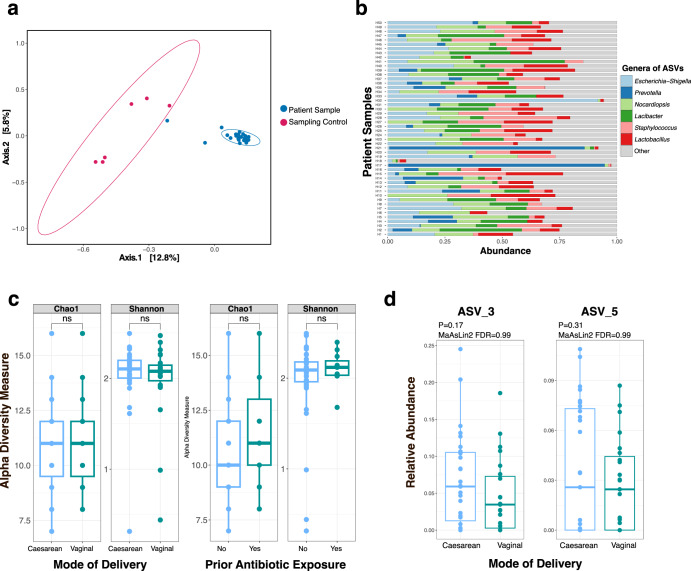
16S rRNA amplicon sequencing reveals a consistent urobiome composition. **a** Beta diversity between infant urine samples and sampling controls. Beta diversity was calculated by the phyloseq “ordinate” function using Bray–Curtis distances. Urine samples were significantly different than sampling controls by permutational multivariate analysis of variance (PERMANOVA, *p* = 0.001). PERMANOVA was calculated using the vegan function “adonis2”. Ellipse depicts a 95% confidence level. **b** ASV-level profiles of urine samples from 50 infants. Urine samples are depicted along the vertical axis and relative abundance is on the *x*-axis. Plot created with the microViz function “comp_barplot”. **c** Alpha diversity metrics (Shannon index and Chao1) between urine from infants born by vaginal delivery vs. Cesarean section (left); and between urine from infants previously exposed to antibiotics vs. antibiotic naïve (right). Alpha diversity was calculated within the phyloseq package using the “plot_richness” function. Alpha diversity was not significantly different between groups by Wilcoxon rank sum test (*p* > 0.05). Box-plot graphs are defined as center line—median; box limits—upper and lower quartiles; whiskers—1.5× interquartile range. **d** Relative abundance of *Lactobacillus* ASVs in urine samples between infants born by vaginal delivery vs. Cesarean section. MaAsLin2 differential abundance *P* values and multiple-testing corrected FDR are depicted. Box-plot graphs are defined as center line—median; box limits—upper and lower quartiles; whiskers—1.5× interquartile range.

Some prior urobiome studies^2,48^ have used agarose gel electrophoresis to determine “negative samples” following 16S rRNA amplification and thus excluded those samples from sequencing. The absence of a band in gel electrophoresis to determine negative samples has a false negative rate of 30% and should be avoided when sampling low biomass environments^49^. We subjected all urine samples to 16S rRNA amplification and sequencing. Every subject’s sample had higher sequencing reads than blank extraction controls (Supplementary Dataset 1). Likewise, sampling controls had consistently higher 16S rRNA reads than extraction blanks. Prior to decontamination, the sampling controls (*n* = 12) clustered distinctly from the subjects’ urine samples (*n* = 50) (PERMANOVA *p* = 0.001) (Fig. 2a). Nonetheless, the number of 16S rRNA reads in sampling controls indicates that this is a potential source of contamination that must be accounted for in urobiome studies. We applied the R package Decontam to remove sequences that were more prevalent in the blank extraction controls, PCR blanks, or sampling controls, compared to the patient urine samples.

### Characterizing the urobiome by amplicon sequencing

Following the above-described filtering steps, 18 specific amplicon sequence variants (ASVs) were retained in the urine samples following filtering using Decontam (Supplementary Dataset 2). Urine samples had a median of 10 unique ASVs (range 8–15). Two ASVs were detected in all 50 subject urine samples and annotated as the following genera: *Nocardiopsis* and *Acinetobacter*. Four ASVs were detected in >45 of the 50 urine samples. These ASVs were annotated as the genera *Staphylococcus*, *Escherichia*-*Shigella*, *Pseudomonas*, and *Nocardiopsis*. The most abundant ASVs were taxonomically annotated as the genera *Escherichia*-*Shigella*, *Prevotella, Nocardiopsis, Lacibacter, Staphylococcus*, and *Lactobacillus* (Fig. 2b).

We sought to investigate whether various subject exposures influenced the diversity of the urobiome. Measures of community diversity are commonly used to summarize information about the richness and distribution of microbials species in the community^50,51^. We compared two measures of alpha diversity (Chao1, Shannon) for two subject exposures: mode of birth (vaginal delivery vs. Cesarean section) and prior antibiotic exposure (Fig. 2c). While both of these exposures are known to alter the gastrointestinal and skin microbiota of infants^52^, no significant difference in alpha diversity was detected in the urine samples between either exposure (Fig. 2c).

Next, we sought to determine whether specific ASVs were influenced by subjects’ exposures. We used MaAsLin2 (Microbiome Multivariable Associations with Linear Models) to perform differential abundance testing^53^. We included delivery mode, age, and prior antibiotic exposure as fixed effects. None of the ASVs were differentially abundant relative to these patient exposures (Supplementary Table 2). For example, two ASVs taxonomically annotated as belonging to the *Lactobacillus* genera (ASV_3 and ASV_5) were present in variable amounts in the 50 subjects. *Lactobacillus* are well-studied members of the urogenital microbiota, particularly in post-pubescent females^8,28,54,55^. *Lactobacillus* are transferred to infants during vaginal birth, and intestinal abundance of *Lactobacillus* is decreased in infants born by Cesarean section^56^. The MaAsLin2 model compared the two *Lactobacillus* ASVs between infants born by vaginal birth *vs*. Cesarean section (Fig. 2d). There was no significant difference in the abundance of these ASVs between these groups. Together, these data display a detectable urobiome among infant males. Six ASVs were detected in ≥45 of the 50 urine samples. Early life exposures, such as mode of birth and prior antibiotic exposure, did not significantly influence urobiome composition.

### Enhanced urine culture identifies culturable members of the infant urobiome

To facilitate future mechanistic studies between urobiome members and the urothelium, or uropathogenic bacteria, we designed an enhanced urine culture protocol with the goal of capturing as many bacteria as possible using limited urine volume from infants. Indeed, 32/50 (64%) of urine samples led to identifiable growth on one or more of the media and conditions. This percentage is consistent with several prior urobiome studies utilizing enhanced culture across the human lifespan^1,2,28,36^. Colony identification was performed by matrix-assisted laser desorption/ionization (MALDI) mass spectrometry. Among the 12 sampling controls, only 1 colony grew from enhanced culture, *Cutibacterium acnes*, a likely skin contaminant. This suggests that the 16S rRNA reads observed in the sampling controls were due to residual DNA, not viable bacteria.

The species identified by enhanced culture, are listed in Table 2. The range of unique species was 1–5 per urine sample. The most common taxonomic families detected were Actinomycetaceae (*n* = 15), Peptoniphilaceae (*n* = 7), and Enterococcaceae (*n* = 6). The most common species isolated were *Actinotignum* spp. (*n* = 9), *Enterococcus faecalis* (*n* = 6), and *Peptoniphilus harei* (*n* = 5).

**Table 2:** Enhanced urine culture results.

Species	Number of isolates
*Actinomyces europaeus*	1
*Actinomyces naeslundii*	1
*Actinomyces odontolyticus*	1
*Actinomyces radingae*	1
*Actinomyces turicensis*	2
*Actinotignum* spp. group	9
*Aerococcus urinae*	1
*Alloscardovia ommnicolens*	1
*Anaerococcus* spps.	1
*Bacillus cereus*	1
*Bifidobacterium breve*	1
*Bifidobacterium dentium*	1
*Bifidobacterium longum*	1
*Citrobacter koseri*	1
*Clostridium sordelli*	1
*Clostridium tertium*	1
*Corynebacterium aurimucosum* group	1
*Corynebacterium* spps.	1
*Cutibacterium acnes*	2
*Enterobacter aerogenes*	1
*Enterococcus faecalis*	6
*Escherichia coli*	2
*Finegoldia magna*	1
*Klebsiella oxytoca*	1
*Murdochiella asaccharolytica*	2
*Paenibacillus* spps.	1
*Peptoniphilus harei*	5
*Peptostreptococcus anaerobius*	1
*Prevotella corporis*	1
*Prevotella* spps.	1
*Prevotella timonensis*	1
*Proteus mirabilis*	1
*Rauotella ornithinolytica*	1
*Rothia aeria*	1
*Staphylococcus aureus*	1
*Staphylococcus capitis*	1

**Fig. 3:**
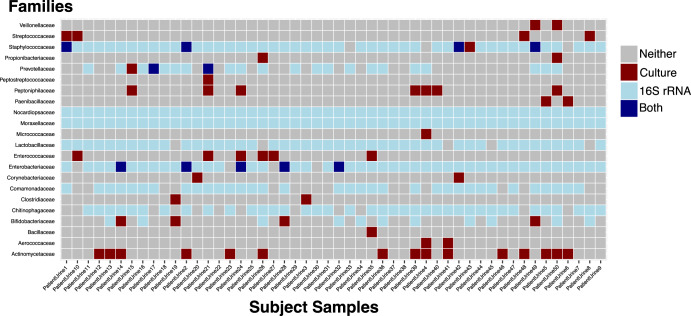
Concordance between enhanced culture and amplicon sequencing results. Co-occurrence detection patterns of taxonomic families between enhanced culture and amplicon sequencing methodologies. Taxonomic families are arranged vertically and patient samples are horizontally. The rectangles indicate the detection of the family by enhanced culture (maroon), 16S rRNA amplicon sequencing (light blue), both methodologies (dark blue), or neither methodology (gray).

Enhanced culture and amplicon sequencing are complementary but not strictly equivalent approaches. We created a concordance map that displays which taxonomic families were detected by enhanced culture, 16S rRNA sequencing, or both (Fig. 3).

We inspected the concordance map for taxonomic families disproportionately represented in either enhanced culture or 16S rRNA results. The families Moraxellaceae, Nocardiopsaceae, and Pseudomonadaceae were detected in most urine samples by 16S rRNA amplicon sequencing, but not by enhanced culture. Similarly, the physiologically important family Lactobacillaceae was frequently detected by amplicon sequencing but not isolated by enhanced culture. The families Enterococcaceae, Peptoniphilaceae, Streptococcaceae were detected >3 times by enhanced culture but were not present in the 16S rRNA results. The absence of these taxonomic families in the 16S rRNA sequencing results indicates shortcomings of the DNA extraction and sequencing methodologies employed in this study. These discordances highlight the potential limitations of each method and the importance of complementary approaches for sampling the urobiome^9,57^.

### *Actinotignum* spp. are a common culturable constituent of the infant urobiome

*Actinotignum* spp. was the most common genus identified in our enhanced culture. Of the 32 urine samples that grew at least one bacterial species, nine (28.1%) grew *Actinotignum* spp. In the 16S rRNA results, ASVs corresponding to the *Actinotignum* genus were removed during filtering as they had relatively low abundance in the dataset as a whole (Supplementary Dataset 1). *Actinotignum schaalii* (formerly *Actinobaculum schaalii*) has been reported in numerous urobiome studies to date^2,3,28,36–40^. Intriguingly, in addition to being reported in this study and others as an asymptomatic colonizer of the urobiome, *A. schaalii* is also an opportunistic causative agent of urinary tract infections^58^. Specifically, there is concern about an increasing incidence of *A. schaalii* urinary tract infections^41–43^. Given the relatively fastidious growth requirements of *A. schaalii*, standard clinical microbiological techniques may not detect *A. schaalii* from urine samples ^43,59,60^, highlighting the need to broaden our understanding of *A. schaalii* in the urinary tract. To date, genomic analysis of *Actinotignum* spp. has been limited to single isolates^61^. To expand our understanding of the *Actinotignum* genus, we performed whole-genome sequencing on nine separate *Actinotignum* isolates identified by enhanced culture of urine from male infants. We generated high-quality whole genome sequences of each *Actinotignum* isolate, with a mean Q30 sequencing coverage of 275x (Supplementary Table 3). The mean genome length was 2,325,278 bp with an average of 1931 coding sequences (CDS).

All nine isolates were identified by MALDI-TOF as “*Actinotignum schaalii*” which is the only *Actinotignum* species present in the MALDI-TOF reference database. Following whole genome sequencing, five of these isolates (AS50, AS1050, AS1053, AS1230, and AS1349) were reclassified as *Actinotignum sanguinis* based on average nucleotide identity. The *Actinotignum* genus is comprised of four species *A. schaalii, A. sanguinis*, *A. timonense*, and *A. urinale*. This misidentification of isolates by MALDI-TOF highlights the limited genomic knowledge of this genus.

**Fig. 4:**
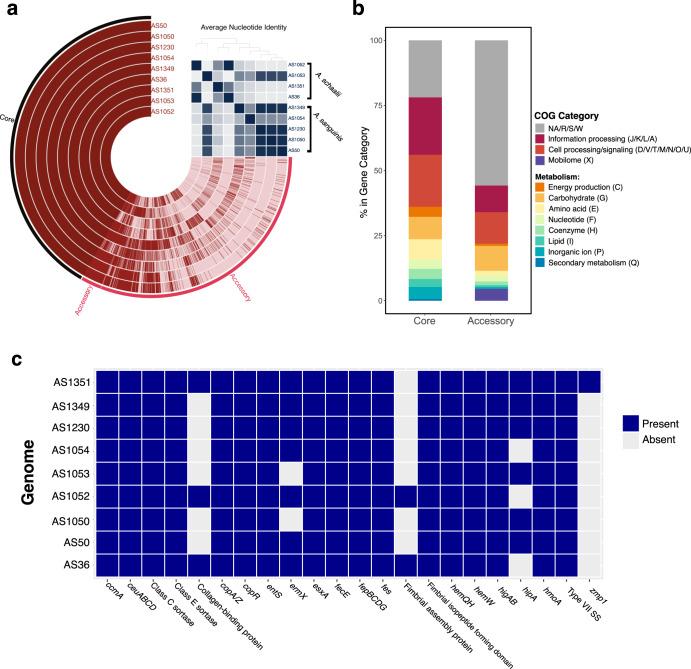
Genomic characterization of *Actinotignum* spp. isolates. **a** Nine *Actinotignum* spp. genomes isolated by enhanced culture from distinct subjects were subjected to whole genome sequencing. *Actinotignum* spp. pangenome of the nine isolates visualized using anvi’o. Core genes were present in 100% of isolates (9/9) while the accessory genome consists of genes present in <9 of the genomes. Clustering of the genomes is based on average nucleotide identity (ANI), shown in the upper right matrix. **b** Relative abundance of COG categories represented in the core and accessory genomes. **c** Presence–absence matrix of fitness factors and antimicrobial resistance genes. ABRicate was used to screen contigs using the MegaRes, ResFinder, and Virulence Factor databases.

Following the annotation of genes with Bakta^62^, we computed the pangenome with Roary^63^. The core genome shared by all nine isolates was composed of 831 genes. An additional 2081 genes were found in 2–8 of the isolates. Finally, there were 1626 unique genes found in only 1 of the nine isolates. We visualized the pangenome and calculated average nucleotide identify (ANI) with anvi’o (Fig. 4a). We compared the gene clusters in the core and accessory genomes using by annotating clusters by COG category within anvi’o. Overall, 21.9% of the core genome and 55.8% of the accessory genome were classified as general functions (*R*), unknown functions (*S*) or unassigned within the COG database (NA) (Fig. 4b). The core genome was enriched for genes involved in information processing (DNA replication, transcription, etc.; COG J/K/L/A), cell processing/signaling (COG D/V/T/M/N/O/U), and energy production (COG C). Interestingly the accessory genome was enriched for genes involved in carbohydrate metabolism (COG G) Intuitively, gene clusters involved in mobile gene transfer (COG X) were elevated in the accessory genome (4.6% vs. 0.06%), consistent with the flexible nature of the accessory genome.

To identify potential determinants of *Actinotignum* spp. fitness in the urinary tract, we used ABRicate to screen for the presence of antimicrobial resistance genes and known fitness factors, utilizing the ResFinder, MegaRes, and the VirulenceFinder Database. Notably, the Actinomycetaceae family is poorly represented in the VFDB and genomic datasets in general^64^, limiting the identification of putative virulence factors. We also annotated contigs with Bakta which reduces the number of CDS annotated as hypothetical proteins^62^. We manually curated potential fitness factors from the Bakta annotations. Seven of the nine isolates encoded *ermX*, an rRNA methyltransferase conferring resistance to macrolides. All nine isolates encoded the Esx-1 Type VII secretion system and its toxin *esxA* (Fig. 4c). Esx-1 has been most extensively characterized in *Mycobacterium tuberculosis*, a member of the phylum Actinobacteria like *A. schaalii*^65,66^. EsxA is an anti-eukaryotic membrane-permeabilizing toxin and is required for virulence in *M. tuberculosis*^66^.

Metal acquisition and homeostasis are key fitness determinants for microbial-host interactions^67,68^. All nine isolates contained the enterobactin transporters, *entS* and *fepBCDG* (Fig. 4c). The siderophore enterobactin, an iron-chelating small molecule, is a known fitness factor within the iron-deplete urinary tract^69^. Analysis of assembled contigs using antiSMASH^70,71^ to identify biosynthetic gene clusters (BGCs), particularly those responsible for siderophores production, did not reveal any putative BGCs that may produce enterobactin or related molecules (Supplementary Table 4). Systems for the acquisition and metabolism of heme were also ubiquitous in the 9 isolates. Specifically, all nine isolates encoded *hemQ* and *hemH* (coproheme decarboxylase and ferrochetalase, respectively) which are involved in heme biosynthesis, the heme chaperone *hemW*, the heme ATPase transporter *ccmA*, and the heme-degrading monooxygenase *hmoA*. Furthermore, all nine isolates encoded copper detoxification systems. Copper is toxic to bacteria in high concentrations and is elevated in the urinary tract during infection^72,73^. Thus, copper detoxification is considered a fitness factor in the urinary tract. All nine isolates encoded *copA/Z*, a copper exporter and chaperone respectively, and *copR*, a copper-responsive transcriptional regulator (Fig. 4c). Together, these results indicate that *Actinotignum* spp. encode known fitness factors within the phylum Actinobacteria (e.g. EsxA) and within disparately related urinary pathogens (e.g. metal acquisition and detoxification).

## Discussion

The importance of understanding how the microbiome of a given anatomic niche shapes the biology and health of a host has never been more critical. In the genitourinary tract, it is now well-accepted that a urobiome exists and plays a role in several urologic conditions^7,11,20,21,74^. Yet, compared to other anatomic niches, like the oral cavity, the gut, and the skin, research surrounding the microbiome of the bladder is in its infancy. We use complementary approaches of enhanced culture and 16S rRNA amplicon sequencing to identify bacteria in catheterized urine samples from healthy infant males. With enhanced culture, we isolated 43 unique bacterial species and 64% of urine samples grew at least one colony by enhanced culture (Table 2), a percentage consistent with many prior urobiome studies^1,2,28,36^. These patient-derived isolates open exciting avenues for studying the interactions of urobiome members.

Our study reveals six ASVs (taxonomically annotated as *Staphylococcus*, *Nocardiopsis*, *Acinetobacter*, *Pseudomonas*, *Escherichia*-*Shigella*, *Lactobacillus*) that were detected by 16S rRNA amplicon sequencing in >45 of the 50 urine samples. This suggests a consistent urobiome composition among healthy infant males. Urobiome alpha diversity was not significantly different between infants born by vaginal vs. Cesarean section, nor was diversity affected by prior antibiotic exposure (Fig. 2c).

Males younger than one-year-old have higher rates of urinary tract infection (UTI) than females^75^. Various explanations for this difference have been proposed, including hormone levels and lack of circumcision^76,77^. The abundance of the genus *Escherichia-Shigella*, the predominant cause of UTIs, in our dataset (Fig. 2b) may offer an additional exploratory hypothesis for the higher rates of UTIs in male infants. As previously noted, none of the subjects in this study had a prior UTI. Notably, in our cohort, urobiome alpha diversity was not significantly different between infants born by vaginal vs. Cesarean section, nor was diversity affected by prior antibiotic exposure (Fig. 2c). This could be due to several reasons: the median age of the male subjects was 7 months old; it is possible that any differences in urobiome composition arising from different modes of delivery have not persisted over time. Another possibility is that once the urobiome is established, it is not perturbed by diet, given the limited metabolites that are excreted in the urine compared to the gut. Likewise, depending on the antibiotic class, dosage, and duration of course, antibiotic concentrations in the urine may not have been sufficient to leave a lasting imprint on the urobiome.

Interestingly, amplicon sequencing did not detect the genus *Porphyromonas* in high abundance, nor did any samples grow *Porphyromonas* on enhanced culture. *Porphyromonas* has been previously described as a major component of the male pediatric urobiome from voided urine samples^32^. Our analysis and another pediatric urobiome study^78^ did not detect *Porphyromonas* in catheterized samples, suggesting that *Porphyromonas* may originate from the urethra and not the bladder. This observation is supported by urethra-specific sampling in adult males^79^.

The genus *Nocardiopsis*, was frequently identified in our 16S rRNA data (Figs. 2 and 3). The closely related genus *Nocardioides* has been detected in several urobiome studies^48,80^. Still, soil and water bacteria, like *Nocardiopsis*, are well-described contaminants of laboratory supplies and reagents. Our methods for filtering contaminants did not remove *Nocardiopsis* from our subjects’ samples. This requires further attention to determine whether *Nocardiopsis* may be a yet unculturable member of the urobiome or an unfiltered sequencing contaminant.

As ongoing research continues to define the urinary microbiome, standardized methods and reporting are vital^44^. Given the low biomass of the urinary microbiome, the potential for contamination is high. We utilized rigorous sampling and processing controls to benchmark the potential contaminants introduced during sample collection and processing. Our inclusion of sampling controls mirrors several other studies of the urobiome and emphasizes the importance of this practice going forward^1,4,81^. Following the sampling of more catheter types and collection environments, the contaminant identification package SourceTracker may become useful within the urobiome field^82^. The analysis of urobiome amplicon sequencing data must account for the low biomass of this sample type and potential sources of contamination. Thus far, reporting of analysis parameters and filtering thresholds has been insufficient for the replication of these studies. To promote reproducibility within the urobiome field, we have included extensive methodological and bioinformatic detail herein. We hope this resource will improve the reproducibility of amplicon sequencing analysis by the urobiome field.

Finally, thus far, urobiome studies have been predominately descriptive studies. Using *Actinotignum* spp. as representative examples, we show how the complementary approaches of enhanced culture and sequencing can uncover exploratory hypotheses by which bacteria may colonize and opportunistically infect the urinary tract. Using whole genome sequencing of nine *Actinotignum* spp. genomes isolated by enhanced culture, we identified that *Actinotignum* spp. possess the transporters for enterobactin uptake but not the biosynthetic machinery for its production. This intriguing observation raises the question of whether *Actinotignum* spp. produce yet unidentified siderophores or whether *Actinotignum* spp. may utilize siderophores produced by other urobiome constituents, known as xenosiderophore scavenging. These observations produce a testable hypothesis for the interactions of *Actinotignum* spp. with other members of the urobiome community.

Our study has several limitations. As noted above, discrepancies between 16S rRNA and enhanced culture results point to the relative strengths and weaknesses of each method. Further methodological refinement of culture techniques and DNA extraction from urine may yield more consistent results in future studies. Our study is limited by its taxonomic resolution which was not able to resolve species-level classifications. Sequencing of larger segments of the 16S rRNA gene or shotgun metagenomic sequencing would add taxonomic granularity to urobiome results. Next, our sample size of 50 infants may have been insufficient to distinguish small differences between exposure histories. Finally, currently, available differential abundance testing methods are neither developed nor optimized for low-biomass samples such as urine. Thus, these methods may be inadequate to properly distinguish differentially abundant sequencing features.

In summary, our study provides a snapshot of the pediatric urobiome of healthy infant males. From enhanced culture, we create an inventory of cultured urobiome constituents for future mechanistic studies. Finally, we report a comprehensive map of genomic features for the urobiome resident genus *Actinotignum* which appears to exhibit both commensal and uropathogenic properties in humans.

## Methods

### Recruitment and sample collection

This study was approved by the Vanderbilt University Medical Center Institutional Review Board (IRB # 191815). Parental guardians provided written informed consent for sterile urinary catheterization under anesthesia in the operating room prior to the circumcision procedure. Exclusion criteria included structural or functional genitourinary abnormalities, prior urinary tract infection, or prior urethral catheterization. Urine samples were collected by transurethral catheterization following sterilization of the glans penis and foreskin. Urine samples were stored in sterile Falcon tubes and immediately placed on ice for transport to permanent storage. Within 2 h of collection, urine samples were transferred to an −80 °C freezer for indefinite storage.

### Enhanced urine culture

Prior to freezing urine samples, 100 μL of urine was spread onto Columbia Agar with 5% Sheep Blood (BD BBL™ 221263) and Brucella Agar (Thermo Scientific™ R01255). Duplicate plates were incubated in either an ambient atmosphere with 5% CO_2_ supplementation or anaerobic conditions (anaerobiosis was attained using BD GasPak™ EZ anaerobe pouch system). Negative control plates of each respective agar were incubated simultaneously. Plates were incubated for up to 5 days at 37 °C. Colonies were analyzed by MALDI-TOF (Bruker Daltonics), and colony identification was performed pyrochemically by MALDI Biotyper® (Bruker Corporation) with an extended research-use taxonomic library. Glycerol stocks were frozen for each unique colony isolated.

### Amplicon 16S rRNA sequencing

Urine samples were shipped on ample dry ice to the University of California at San Diego Microbiome Center for DNA extraction and sequencing. DNA was extracted using the ThermoFisher MagMAX™ Microbiome Ultra Nucleic Acid Isolation Kit (A42357) from 500 μL of sample. To benchmark DNA extraction efficiency, ten-fold serial dilutions of ZymoBIOMICS™ Microbial Community Standard (D6300) were extracted in parallel with urine samples. Following DNA extraction, the V4 hypervariable 16S rRNA region was amplified using the 515F and 806R primers from the Earth Microbiome Project^83^. To benchmark PCR amplification of 16S rRNA, ten-fold serial dilutions of ZymoBIOMICS™ Microbial Community DNA Standard (D6306) were amplified in parallel with extraction standards and urine samples. Additionally, negative control wells (extraction blanks) lacking sample input were subjected to DNA extraction, PCR amplification, and sequencing. Further description of the methods and controls is available in the Supplementary Methods.

### 16S rRNA sequencing analysis

All sequencing processing and analyses were completed in R (version 4.2.1). Sequences were processed using DADA2 to trim, filter, learn error rates, denoise, merge, and remove chimeras from reads^84^. Amplicon sequence variants (ASVs) were assigned to merged reads with the SILVA rRNA database (version 138.1) using the DADA2 function assignTaxonomy. ASVs were merged with their taxonomy in the R package phyloseq^85^. The R package Decontam was used to identify and remove potential contaminant ASVs using the prevalence method and a threshold of 0.4. Furthermore, ASVs <1% abundance in the entire dataset were excluded. These thresholds were chosen after evaluating the removal of contaminating ASVs of the ZymoBIOMICS™ Microbial Community Standard dilution series (Supplementary Methods). Similar thresholds have previously been applied to urobiome datasets^74^. The R packages microbiome, microViz, and vegan were used to format and visualize the 16S rRNA data. Differential abundance testing of ASVs was conducted with MaAsLin2 (Microbiome Multivariable Associations with Linear Models) using the delivery mode, age, and prior antibiotic exposure as fixed effects in a linear model^53^. Multiple-testing-corrected *P* values are described as false disovery rates (FDR).

### DNA extraction from *Actinotignum* spp. and whole genome sequencing

Colonies of *Actinotigum* spp. were resuspended in PBS, lysed with a combination of lysostaphin, lysozyme, and Proteinase K at 37 °C. Next, the suspension was treated with RNase. Following dilution of the lysate in H_2_O, the mixture was sonicated at 34 kHz for 4 min. DNA was purified with three successive extractions in phenol:chloroform:isoamyl alcohol (25:24:1). Finally, DNA was precipitated from ethanol and resuspended in H_2_O. DNA was sent on dry ice to SeqCenter (formerly, Microbial Genomic Sequencing Center, Pittsburgh, PA). Sample libraries were prepared using the Illumina DNA Prep kit and IDT 10 bp UDI indices, and sequenced on an Illumina NextSeq 2000, producing 2 × 151 bp reads. Demultiplexing, quality control, and adapter trimming were performed with bcl-convert (v3.9.3).

### *Actinotignum* spp. sequencing analysis

Trimmed reads were assembled into contigs >1000 bp using Shovill (v. 1.1)^86^. Contigs were annotated with Bakta using default settings^62^. Annotated files were analyzed by Roary to construct the core genome and pangenome^63^. Anvi’o was used to visualize the pangenome, calculate average nucleotide identity, and visualize a phylogenetic tree^87,88^. Contigs were reformatted into anvi’o format and annotated with the COG20 database^89^. Functional enrichment of the core and accessory genomes was calculated using anvi’o pangenome summarize function. ABRicate was used to determine the presence of antimicrobial resistance genes and virulence factors on the unannotated contigs. ResFinder (v. 4.0), MegaRes (v. 3.0), and VirulenceFinder Database (VFDB, v. 5). were used as the reference databases of ABRicate. Contigs were uploaded to the antiSMASH online interface and analyzed with antiSMASH beta version 7.0 which includes an updated algorithm for the prediction of non-ribosomal peptide-produced metallophores^70,71^. AntiSMASH settings were relaxed detection strictness, KnownClusterBLAST, MIBig cluster comparison^90^, and Cluster Pfam analysis.

### Reporting summary

Further information on research design is available in the Nature Research Reporting Summary linked to this article.

### Supplementary information

Supplementary file

Supplementary Data 1

Supplementary Data 2

Reporting Summary

## Data Availability

All sequence data derived from this work are publicly available in NCBI-Genbank databases under Bioproject PRJNA912725. NCBI-Genbank accession numbers are listed within Supplementary Table 5.

## Code availability

All code used for bioinformatic analysis is publicly available within the Supplementary Methods and online at https://github.com/reaset41/Infant-Urobiome.
