# Supplementary material for: Survey of the infant male urobiome and genomic analysis of Actinotignum spp
Source: NPJ Biofilms Microbiomes. 2023 Dec 1;9:91. doi: 10.1038/s41522-023-00457-6 (PMC10692110; doi:10.1038/s41522-023-00457-6)
Supplement: Supplementary file 1 — Supplementary file [file 41522_2023_457_MOESM1_ESM.pdf]

SUPPLEMENTARY INFORMATION

Supplementary Table 1. Complete Metadata for All Samples

Supplementary Table 2. MaAsLin2 Differential Abundance Testing of ASVs

Supplementary Table 3. *Actinotignum* spp. Genome Assembly Statistics

Supplementary Table 4. Biosynthetic Gene Clusters from antiSMASH

Supplementary Table 5. Accession Information for BioProject PRJNA912725

Supplementary Methods. Bioinformatic Code

Supplementary Table 1. Complete Metadata for All Samples

SampleID	Biosample	SampleType	SequencingPlatform	Age_at_collection_days	Race	NICU	PriorAbx	Preterm_Term_delivery	Delivery_M	Nutrition	Volume_urine_mL	is.neg
PatientUrine1	SAMN33342268	PATIENT	MiSeq	215	White	No	No	Fullterm	Caesarean	Formula	4.4	FALSE
PatientUrine10	SAMN33342277	PATIENT	MiSeq	319	White	No	Yes	Preterm	Caesarean	Formula	1	FALSE
PatientUrine11	SAMN33342278	PATIENT	MiSeq	241	White	No	No	Preterm	Vaginal	Formula	6	FALSE
PatientUrine12	SAMN33342279	PATIENT	MiSeq	183	African-American	No	No	Fullterm	Caesarean	BreastMilk	6	FALSE
PatientUrine13	SAMN33342280	PATIENT	MiSeq	317	White	No	Yes	Fullterm	Caesarean	Formula/Solids	16	FALSE
PatientUrine14	SAMN33342281	PATIENT	MiSeq	228	White	No	No	Preterm	Caesarean	BreastMilk	3.9	FALSE
PatientUrine15	SAMN33342282	PATIENT	MiSeq	189	African-American	Yes	No	Fullterm	Caesarean	BreastMilk/Formula	1.5	FALSE
PatientUrine16	SAMN33342283	PATIENT	MiSeq	261	White	No	No	Fullterm	Caesarean	BreastMilk	4.4	FALSE
PatientUrine17	SAMN33342284	PATIENT	MiSeq	201	White	No	No	Fullterm	Caesarean	Formula	3	FALSE
PatientUrine18	SAMN33342285	PATIENT	MiSeq	185	African-American	No	No	Fullterm	Vaginal	BreastMilk/Solids	4	FALSE
PatientUrine19	SAMN33342286	PATIENT	MiSeq	194	White	Yes	No	Fullterm	Vaginal	BreastMilk	8	FALSE
PatientUrine2	SAMN33342269	PATIENT	MiSeq	226	African-American	Yes	No	Fullterm	Caesarean	Formula	9.4	FALSE
PatientUrine20	SAMN33342287	PATIENT	MiSeq	183	Asian	No	No	Fullterm	Vaginal	BreastMilk/Formula	0.4	FALSE
PatientUrine21	SAMN33342288	PATIENT	MiSeq	344	White	Yes	Yes	Fullterm	Caesarean	BreastMilk/Formula	4	FALSE
PatientUrine22	SAMN33342289	PATIENT	MiSeq	227	White	No	No	Preterm	Caesarean	Formula	1.5	FALSE
PatientUrine23	SAMN33342290	PATIENT	MiSeq	189	African-American	No	No	Fullterm	Vaginal	Formula	3	FALSE
PatientUrine24	SAMN33342291	PATIENT	MiSeq	189	2 or more races/other	Yes	No	Fullterm	Vaginal	BreastMilk/Formula	5	FALSE
PatientUrine25	SAMN33342292	PATIENT	MiSeq	219	White	No	No	Fullterm	Caesarean	Formula	8	FALSE
PatientUrine26	SAMN33342293	PATIENT	MiSeq	221	2 or more races/other	No	No	Preterm	Caesarean	BreastMilk/Formula	6	FALSE
PatientUrine27	SAMN33342294	PATIENT	MiSeq	204	2 or more races/other	Yes	No	Fullterm	Caesarean	BreastMilk/Formula	18	FALSE
PatientUrine28	SAMN33342295	PATIENT	MiSeq	208	2 or more races/other	Yes	Yes	Fullterm	Vaginal	Formula	2	FALSE
PatientUrine29	SAMN33342296	PATIENT	MiSeq	188	African-American	No	No	Fullterm	Vaginal	BreastMilk/Formula	8.5	FALSE
PatientUrine3	SAMN33342270	PATIENT	MiSeq	348	White	Yes	No	Fullterm	Vaginal	Formula	28	FALSE
PatientUrine30	SAMN33342297	PATIENT	MiSeq	250	White	No	No	Preterm	Vaginal	Formula/Solids	3	FALSE
PatientUrine31	SAMN33342298	PATIENT	MiSeq	194	African-American	No	No	Fullterm	Vaginal	Formula	6	FALSE
PatientUrine32	SAMN33342299	PATIENT	MiSeq	237	White	Yes	No	Preterm	Vaginal	Formula/Solids	0.8	FALSE
PatientUrine33	SAMN33342300	PATIENT	MiSeq	190	Pacific Islander	No	No	Preterm	Caesarean	BreastMilk/Formula	11	FALSE
PatientUrine34	SAMN33342301	PATIENT	MiSeq	190	African-American	Yes	No	Preterm	Caesarean	BreastMilk/Formula	5.8	FALSE
PatientUrine35	SAMN33342302	PATIENT	MiSeq	190	African-American	Yes	No	Fullterm	Caesarean	BreastMilk/Formula	1	FALSE
PatientUrine36	SAMN33342303	PATIENT	MiSeq	258	African-American	No	No	Fullterm	Vaginal	BreastMilk/Formula	2	FALSE
PatientUrine37	SAMN33342304	PATIENT	MiSeq	185	White	No	No	Fullterm	Caesarean	BreastMilk	2	FALSE
PatientUrine38	SAMN33342305	PATIENT	MiSeq	216	White	Yes	Yes	Preterm	Vaginal	BreastMilk/Formula	8.5	FALSE
PatientUrine39	SAMN33342306	PATIENT	MiSeq	213	White	No	No	Fullterm	Vaginal	Formula/Solids	1.5	FALSE
PatientUrine4	SAMN33342271	PATIENT	MiSeq	320	African-American	No	Yes	Preterm	Caesarean	Formula/Solids	7	FALSE
PatientUrine40	SAMN33342307	PATIENT	MiSeq	319	White	No	Yes	Preterm	Caesarean	BreastMilk/Formula	1.5	FALSE
PatientUrine41	SAMN33342308	PATIENT	MiSeq	214	Asian	No	No	Fullterm	Caesarean	Solids	3	FALSE
PatientUrine42	SAMN33342309	PATIENT	MiSeq	180	White	Yes	No	Fullterm	Vaginal	BreastMilk/Solids	4.5	FALSE
PatientUrine43	SAMN33342310	PATIENT	MiSeq	247	2 or more races/other	No	No	Preterm	Caesarean	Solids	8.5	FALSE
PatientUrine44	SAMN33342311	PATIENT	MiSeq	188	2 or more races/other	No	No	Fullterm	Caesarean	BreastMilk/Formula	0.7	FALSE
PatientUrine45	SAMN33342312	PATIENT	MiSeq	270	White	Yes	Yes	Preterm	Vaginal	BreastMilk/Formula	3	FALSE
PatientUrine46	SAMN33342313	PATIENT	MiSeq	195	2 or more races/other	Yes	No	Fullterm	Vaginal	BreastMilk	16	FALSE
PatientUrine47	SAMN33342314	PATIENT	MiSeq	189	White	No	No	Preterm	Vaginal	BreastMilk/Formula	1.7	FALSE
PatientUrine48	SAMN33342315	PATIENT	MiSeq	203	White	No	No	Fullterm	Caesarean	Formula/Solids	5	FALSE
PatientUrine49	SAMN33342316	PATIENT	MiSeq	203	White	Yes	No	Fullterm	Caesarean	Formula/Solids	6.5	FALSE
PatientUrine5	SAMN33342272	PATIENT	MiSeq	285	White	No	Yes	Preterm	Vaginal	Formula	9	FALSE
PatientUrine50	SAMN33342317	PATIENT	MiSeq	310	African-American	Yes	Yes	Fullterm	Vaginal	BreastMilk/Solids		FALSE
PatientUrine6	SAMN33342273	PATIENT	MiSeq	241	White	Yes	No	Fullterm	Vaginal	Formula	9.1	FALSE
PatientUrine7	SAMN33342274	PATIENT	MiSeq	252	African-American	No	No	Fullterm	Vaginal	Solids	12.1	FALSE
PatientUrine8	SAMN33342275	PATIENT	MiSeq	220	White	No	No	Preterm	Caesarean	BreastMilk/Solids	9.5	FALSE
PatientUrine9	SAMN33342276	PATIENT	MiSeq	319	White	No	Yes	Preterm	Caesarean	Formula	3.5	FALSE
SamplingControl1	SAMN33342338	SamplingCon	NovaSeq	NA	NA	NA	NA	NA	NA		NA	TRUE
SamplingControl2	SAMN33342339	SamplingCon	NovaSeq	NA	NA	NA	NA	NA	NA		NA	TRUE
SamplingControl3	SAMN33342340	SamplingCon	NovaSeq	NA	NA	NA	NA	NA	NA		NA	TRUE
SamplingControl4	SAMN33342341	SamplingCon	NovaSeq	NA	NA	NA	NA	NA	NA		NA	TRUE
SamplingControl5	SAMN33342342	SamplingCon	NovaSeq	NA	NA	NA	NA	NA	NA		NA	TRUE
SamplingControl6	SAMN33342343	SamplingCon	NovaSeq	NA	NA	NA	NA	NA	NA		NA	TRUE
SamplingControl7	SAMN33342344	SamplingCon	NovaSeq	NA	NA	NA	NA	NA	NA		NA	TRUE
SamplingControl8	SAMN33342345	SamplingCon	NovaSeq	NA	NA	NA	NA	NA	NA		NA	TRUE
SamplingControl9	SAMN33342346	SamplingCon	NovaSeq	NA	NA	NA	NA	NA	NA		NA	TRUE
SamplingControl10	SAMN33342347	SamplingCon	NovaSeq	NA	NA	NA	NA	NA	NA		NA	TRUE
SamplingControl11	SAMN33342348	SamplingCon	NovaSeq	NA	NA	NA	NA	NA	NA		NA	TRUE
SamplingControl12	SAMN33342349	SamplingCon	NovaSeq	NA	NA	NA	NA	NA	NA		NA	TRUE
NovaSeq_PCRBlank1	SAMN33342326	Blank	NovaSeq	NA	NA	NA	NA	NA	NA		NA	TRUE
NovaSeq_PCRBlank2	SAMN33342327	Blank	NovaSeq	NA	NA	NA	NA	NA	NA		NA	TRUE
NovaSeq_PCRBlank3	SAMN33342328	Blank	NovaSeq	NA	NA	NA	NA	NA	NA		NA	TRUE
NovaSeq_PCRBlank4	SAMN33342329	Blank	NovaSeq	NA	NA	NA	NA	NA	NA		NA	TRUE
NovaSeq_ExtractionBlank1	SAMN33342318	Blank	NovaSeq	NA	NA	NA	NA	NA	NA		NA	TRUE
NovaSeq_ExtractionBlank2	SAMN33342319	Blank	NovaSeq	NA	NA	NA	NA	NA	NA		NA	TRUE
NovaSeq_ExtractionBlank3	SAMN33342320	Blank	NovaSeq	NA	NA	NA	NA	NA	NA		NA	TRUE
NovaSeq_ExtractionBlank4	SAMN33342321	Blank	NovaSeq	NA	NA	NA	NA	NA	NA		NA	TRUE
NovaSeq_ExtractionBlank5	SAMN33342322	Blank	NovaSeq	NA	NA	NA	NA	NA	NA		NA	TRUE
NovaSeq_ExtractionBlank6	SAMN33342323	Blank	NovaSeq	NA	NA	NA	NA	NA	NA		NA	TRUE
NovaSeq_ExtractionBlank7	SAMN33342324	Blank	NovaSeq	NA	NA	NA	NA	NA	NA		NA	TRUE
NovaSeq_ExtractionBlank8	SAMN33342325	Blank	NovaSeq	NA	NA	NA	NA	NA	NA		NA	TRUE
NovaSeq_ZymoMockA	SAMN33342330	Mock	NovaSeq	NA	NA	NA	NA	NA	NA		NA	FALSE
NovaSeq_ZymoMockB	SAMN33342331	Mock	NovaSeq	NA	NA	NA	NA	NA	NA		NA	FALSE
NovaSeq_ZymoMockC	SAMN33342332	Mock	NovaSeq	NA	NA	NA	NA	NA	NA		NA	FALSE
NovaSeq_ZymoMockD	SAMN33342333	Mock	NovaSeq	NA	NA	NA	NA	NA	NA		NA	FALSE
NovaSeq_ZymoMockE	SAMN33342334	Mock	NovaSeq	NA	NA	NA	NA	NA	NA		NA	FALSE
NovaSeq_ZymoMockF	SAMN33342335	Mock	NovaSeq	NA	NA	NA	NA	NA	NA		NA	FALSE
NovaSeq_ZymoMockG	SAMN33342336	Mock	NovaSeq	NA	NA	NA	NA	NA	NA		NA	FALSE
NovaSeq_ZymoMockH	SAMN33342337	Mock	NovaSeq	NA	NA	NA	NA	NA	NA		NA	FALSE
MiSeq_ExtractionBlank1	SAMN33342248	Blank	MiSeq	NA	NA	NA	NA	NA	NA		NA	TRUE
MiSeq_ExtractionBlank2	SAMN33342249	Blank	MiSeq	NA	NA	NA	NA	NA	NA		NA	TRUE
MiSeq_ExtractionBlank3	SAMN33342250	Blank	MiSeq	NA	NA	NA	NA	NA	NA		NA	TRUE
MiSeq_ExtractionBlank4	SAMN33342251	Blank	MiSeq	NA	NA	NA	NA	NA	NA		NA	TRUE
MiSeq_ExtractionBlank5	SAMN33342252	Blank	MiSeq	NA	NA	NA	NA	NA	NA		NA	TRUE
MiSeq_ExtractionBlank6	SAMN33342253	Blank	MiSeq	NA	NA	NA	NA	NA	NA		NA	TRUE
MiSeq_ExtractionBlank7	SAMN33342254	Blank	MiSeq	NA	NA	NA	NA	NA	NA		NA	TRUE
MiSeq_ExtractionBlank8	SAMN33342255	Blank	MiSeq	NA	NA	NA	NA	NA	NA		NA	TRUE
MiSeq_PCRBlank1	SAMN33342256	Blank	MiSeq	NA	NA	NA	NA	NA	NA		NA	TRUE
MiSeq_PCRBlank2	SAMN33342257	Blank	MiSeq	NA	NA	NA	NA	NA	NA		NA	TRUE
MiSeq_PCRBlank3	SAMN33342258	Blank	MiSeq	NA	NA	NA	NA	NA	NA		NA	TRUE
MiSeq_PCRBlank4	SAMN33342259	Blank	MiSeq	NA	NA	NA	NA	NA	NA		NA	TRUE
MiSeq_ZymoMockA	SAMN33342260	Mock	MiSeq	NA	NA	NA	NA	NA	NA		NA	FALSE
MiSeq_ZymoMockB	SAMN33342261	Mock	MiSeq	NA	NA	NA	NA	NA	NA		NA	FALSE
MiSeq_ZymoMockC	SAMN33342262	Mock	MiSeq	NA	NA	NA	NA	NA	NA		NA	FALSE
MiSeq_ZymoMockD	SAMN33342263	Mock	MiSeq	NA	NA	NA	NA	NA	NA		NA	FALSE
MiSeq_ZymoMockE	SAMN33342264	Mock	MiSeq	NA	NA	NA	NA	NA	NA		NA	FALSE
MiSeq_ZymoMockF	SAMN33342265	Mock	MiSeq	NA	NA	NA	NA	NA	NA		NA	FALSE
MiSeq_ZymoMockG	SAMN33342266	Mock	MiSeq	NA	NA	NA	NA	NA	NA		NA	FALSE
MiSeq_ZymoMockH	SAMN33342267	Mock	MiSeq	NA	NA	NA	NA	NA	NA		NA	FALSE

Supplementary Table 2. MaAsLin2 Differential Abundance Testing of ASVs

feature	metadata	value	coef	stderr	N	N.not.0	pval	qval
ASV_1	DeliveryMode	Vaginal	0.00034782	0.023954849	50	46	0.98847808	0.98847808
ASV_1	PriorAbx	Yes	0.01762652	0.039608857	50	46	0.65839638	0.98847808
ASV_1	Age_days	Age_days	-0.0212934	0.016633831	50	46	0.20691916	0.98847808
ASV_2	DeliveryMode	Vaginal	0.05287886	0.043385386	50	46	0.22912726	0.98847808
ASV_2	PriorAbx	Yes	-0.0299716	0.071736857	50	46	0.67803781	0.98847808
ASV_2	Age_days	Age_days	0.00830569	0.030126058	50	46	0.78401497	0.98847808
ASV_3	DeliveryMode	Vaginal	-0.0230987	0.016741207	50	39	0.17433328	0.98847808
ASV_3	PriorAbx	Yes	-0.0113165	0.027681247	50	39	0.68457172	0.98847808
ASV_3	Age_days	Age_days	0.00174691	0.011624804	50	39	0.88120484	0.98847808
ASV_4	DeliveryMode	Vaginal	-0.0006655	0.008061265	50	11	0.93456519	0.98847808
ASV_4	PriorAbx	Yes	-0.013211	0.013329138	50	11	0.32680209	0.98847808
ASV_4	Age_days	Age_days	0.00563712	0.005597602	50	11	0.31917404	0.98847808
ASV_5	DeliveryMode	Vaginal	-0.0100909	0.009857824	50	33	0.31135611	0.98847808
ASV_5	PriorAbx	Yes	0.01509528	0.016299712	50	33	0.35922393	0.98847808
ASV_5	Age_days	Age_days	-0.0072364	0.006845101	50	33	0.29595791	0.98847808
ASV_6	DeliveryMode	Vaginal	-0.008865	0.021547973	50	11	0.68268591	0.98847808
ASV_6	PriorAbx	Yes	0.02484668	0.035629137	50	11	0.48908133	0.98847808
ASV_6	Age_days	Age_days	0.02565	0.014962538	50	11	0.09320845	0.98847808
ASV_8	DeliveryMode	Vaginal	-0.0005309	0.011895802	50	50	0.96459699	0.98847808
ASV_8	PriorAbx	Yes	0.00667469	0.019669467	50	50	0.73589608	0.98847808
ASV_8	Age_days	Age_days	-0.0017748	0.008260238	50	50	0.83082714	0.98847808
ASV_9	DeliveryMode	Vaginal	0.03243721	0.032424065	50	7	0.32234809	0.98847808
ASV_9	PriorAbx	Yes	0.00212289	0.053612535	50	7	0.9685859	0.98847808
ASV_9	Age_days	Age_days	-0.0180041	0.022514708	50	7	0.42801789	0.98847808
ASV_11	DeliveryMode	Vaginal	-0.0004895	0.015318177	50	50	0.97464778	0.98847808
ASV_11	PriorAbx	Yes	-0.0086576	0.025328294	50	50	0.73404623	0.98847808
ASV_11	Age_days	Age_days	0.00466077	0.010636676	50	50	0.66330751	0.98847808
ASV_12	DeliveryMode	Vaginal	0.00245557	0.010225234	50	48	0.81128277	0.98847808
ASV_12	PriorAbx	Yes	0.00711397	0.016907218	50	48	0.67588605	0.98847808
ASV_12	Age_days	Age_days	-0.0067656	0.007100225	50	48	0.34563504	0.98847808
ASV_13	DeliveryMode	Vaginal	-0.013262	0.0086312	50	49	0.13126292	0.98847808
ASV_13	PriorAbx	Yes	0.02959224	0.014271514	50	49	0.04375083	0.98847808
ASV_13	Age_days	Age_days	-0.0051082	0.005993355	50	49	0.39845988	0.98847808
ASV_14	DeliveryMode	Vaginal	0.01828133	0.02607884	50	31	0.48683273	0.98847808
ASV_14	PriorAbx	Yes	0.02242732	0.043120833	50	31	0.60548532	0.98847808
ASV_14	Age_days	Age_days	-0.0244468	0.018108693	50	31	0.18362111	0.98847808
ASV_15	DeliveryMode	Vaginal	-0.0083639	0.031583625	50	38	0.79233412	0.98847808
ASV_15	PriorAbx	Yes	-0.0208756	0.052222884	50	38	0.69119773	0.98847808
ASV_15	Age_days	Age_days	0.00401547	0.021931121	50	38	0.85552851	0.98847808
ASV_17	DeliveryMode	Vaginal	-0.0038508	0.017816647	50	35	0.82983977	0.98847808
ASV_17	PriorAbx	Yes	-0.0221854	0.029459464	50	35	0.45523761	0.98847808
ASV_17	Age_days	Age_days	0.01883196	0.01237157	50	35	0.13480524	0.98847808
ASV_18	DeliveryMode	Vaginal	-0.0115703	0.016522423	50	34	0.48727933	0.98847808
ASV_18	PriorAbx	Yes	-0.0048421	0.027319491	50	34	0.86009925	0.98847808
ASV_18	Age_days	Age_days	0.0001892	0.011472883	50	34	0.98691369	0.98847808
ASV_19	DeliveryMode	Vaginal	-0.0256135	0.020881315	50	17	0.22620663	0.98847808
ASV_19	PriorAbx	Yes	-0.0144397	0.034526832	50	17	0.67773477	0.98847808
ASV_19	Age_days	Age_days	0.01559211	0.014499623	50	17	0.28782804	0.98847808

Supplementary Table 3. Actinotignum spp. Genome Assembly Statistics

Strain	Species	BioSample	GenBank_Accession	Contigs	N50
AS36	*Actinotignum schaalii*	SAMN33341089	GCA_028864225.1	49	92,344
AS50	*Actinotignum sanguinis*	SAMN33341090	GCA_028864045.1	53	91,643
AS1050	*Actinotignum sanguinis*	SAMN33341091	GCA_028864185.1	67	100,835
AS1052	*Actinotignum schaalii*	SAMN33341092	GCA_028864235.1	58	74,640
AS1053	*Actinotignum sanguinis*	SAMN33341093	GCA_028864205.1	41	98,650
AS1054	*Actinotignum schaalii*	SAMN33341094	GCA_028864145.1	77	45,454
AS1230	*Actinotignum sanguinis*	SAMN33341095	GCA_028864125.1	95	94,433
AS1349	*Actinotignum sanguinis*	SAMN33341096	GCA_028864065.1	53	85,046
AS1351	*Actinotignum schaalii*	SAMN33341097	GCA_028864105.1	38	125,140

Supplementary Table 4. Biosynthetic Gene Clusters from antiSMASH

Genome	Putative BGCs	BGC Type	Most Similar BGC
AS36	1	T3PKS (Type III polyketide synthases)	BGC0000934 (17% similar)
AS50	None	NA	NA
AS1050	None	NA	NA
AS1052	1	T3PKS (Type III polyketide synthases)	BGC0000934 (17% similar)
AS1053	None	NA	NA
AS1054	1	T3PKS (Type III polyketide synthases)	BGC0001083 (9% similar)
AS1230	None	NA	NA
AS1349	None	NA	NA
AS1351	1	T3PKS (Type III polyketide synthases)	BGC0000934 (17% similar)

Supplementary Table 5. Accession Information for BioProject PRJNA912725

BioSample	SRA_Accession	SampleID	Description
SAMN33341089	SRS16799825	AS36	whole genome sequencing of A. schaalli AS36
SAMN33341090	SRS16799826	AS50	whole genome sequencing of A. schaalli AS50
SAMN33341091	SRS16799827	AS1050	whole genome sequencing of A. schaalli AS1050
SAMN33341092	SRS16799828	AS1052	whole genome sequencing of A. schaalli AS1052
SAMN33341093	SRS16799829	AS1053	whole genome sequencing of A. schaalli AS1053
SAMN33341094	SRS16799830	AS1054	whole genome sequencing of A. schaalli AS1054
SAMN33341095	SRS16799831	AS1230	whole genome sequencing of A. schaalli AS1230
SAMN33341096	SRS16799832	AS1349	whole genome sequencing of A. schaalli AS1349
SAMN33341097	SRS16799833	AS1351	whole genome sequencing of A. schaalli AS1351
SAMN33342248	SRR23560255	MiSeq_ExtractionBlank1	DNA extraction kit blank
SAMN33342249	SRR23560254	MiSeq_ExtractionBlank2	DNA extraction kit blank
SAMN33342250	SRR23560241	MiSeq_ExtractionBlank3	DNA extraction kit blank
SAMN33342251	SRR23560230	MiSeq_ExtractionBlank4	DNA extraction kit blank
SAMN33342252	SRR23560195	MiSeq_ExtractionBlank5	DNA extraction kit blank
SAMN33342253	SRR23560184	MiSeq_ExtractionBlank6	DNA extraction kit blank
SAMN33342254	SRR23560170	MiSeq_ExtractionBlank7	DNA extraction kit blank
SAMN33342255	SRR23560159	MiSeq_ExtractionBlank8	DNA extraction kit blank
SAMN33342256	SRR23560223	MiSeq_PCRBlank1	16S rRNA PCR amplification blank
SAMN33342257	SRR23560212	MiSeq_PCRBlank2	16S rRNA PCR amplification blank
SAMN33342258	SRR23560253	MiSeq_PCRBlank3	16S rRNA PCR amplification blank
SAMN33342259	SRR23560250	MiSeq_PCRBlank4	16S rRNA PCR amplification blank
SAMN33342260	SRR23560249	MiSeq_ZymoMockA	Zymo Mock Microbial community (undiluted)
SAMN33342261	SRR23560248	MiSeq_ZymoMockB	Zymo Mock Microbial community (1/10 dilution)
SAMN33342262	SRR23560247	MiSeq_ZymoMockC	Zymo Mock Microbial community (1/100 dilution)
SAMN33342263	SRR23560246	MiSeq_ZymoMockD	Zymo Mock Microbial community (1/1000 dilution)
SAMN33342264	SRR23560245	MiSeq_ZymoMockE	Zymo Mock Microbial community (10^-4 dilution)
SAMN33342265	SRR23560244	MiSeq_ZymoMockF	Zymo Mock Microbial community (10^-5 dilution)
SAMN33342266	SRR23560243	MiSeq_ZymoMockG	Zymo Mock Microbial community (10^-6 dilution)
SAMN33342267	SRR23560242	MiSeq_ZymoMockH	Zymo Mock Microbial community (10^-7 dilution)
SAMN33342268	SRR23560240	PatientUrine1	human infant male urine patient 1
SAMN33342269	SRR23560239	PatientUrine2	human infant male urine patient 2
SAMN33342270	SRR23560238	PatientUrine3	human infant male urine patient 3
SAMN33342271	SRR23560237	PatientUrine4	human infant male urine patient 4
SAMN33342272	SRR23560236	PatientUrine5	human infant male urine patient 5
SAMN33342273	SRR23560235	PatientUrine6	human infant male urine patient 6
SAMN33342274	SRR23560234	PatientUrine7	human infant male urine patient 7
SAMN33342275	SRR23560233	PatientUrine8	human infant male urine patient 8
SAMN33342276	SRR23560232	PatientUrine9	human infant male urine patient 9
SAMN33342277	SRR23560231	PatientUrine10	human infant male urine patient 10
SAMN33342278	SRR23560229	PatientUrine11	human infant male urine patient 11
SAMN33342279	SRR23560204	PatientUrine12	human infant male urine patient 12
SAMN33342280	SRR23560203	PatientUrine13	human infant male urine patient 13
SAMN33342281	SRR23560202	PatientUrine14	human infant male urine patient 14
SAMN33342282	SRR23560201	PatientUrine15	human infant male urine patient 15
SAMN33342283	SRR23560200	PatientUrine16	human infant male urine patient 16
SAMN33342284	SRR23560199	PatientUrine17	human infant male urine patient 17
SAMN33342285	SRR23560198	PatientUrine18	human infant male urine patient 18
SAMN33342286	SRR23560197	PatientUrine19	human infant male urine patient 19
SAMN33342287	SRR23560196	PatientUrine20	human infant male urine patient 20
SAMN33342288	SRR23560194	PatientUrine21	human infant male urine patient 21
SAMN33342289	SRR23560193	PatientUrine22	human infant male urine patient 22
SAMN33342290	SRR23560192	PatientUrine23	human infant male urine patient 23
SAMN33342291	SRR23560191	PatientUrine24	human infant male urine patient 24
SAMN33342292	SRR23560190	PatientUrine25	human infant male urine patient 25
SAMN33342293	SRR23560189	PatientUrine26	human infant male urine patient 26
SAMN33342294	SRR23560188	PatientUrine27	human infant male urine patient 27
SAMN33342295	SRR23560187	PatientUrine28	human infant male urine patient 28
SAMN33342296	SRR23560186	PatientUrine29	human infant male urine patient 29
SAMN33342297	SRR23560185	PatientUrine30	human infant male urine patient 30
SAMN33342298	SRR23560183	PatientUrine31	human infant male urine patient 31
SAMN33342299	SRR23560182	PatientUrine32	human infant male urine patient 32
SAMN33342300	SRR23560181	PatientUrine33	human infant male urine patient 33
SAMN33342301	SRR23560177	PatientUrine34	human infant male urine patient 34
SAMN33342302	SRR23560176	PatientUrine35	human infant male urine patient 35
SAMN33342303	SRR23560175	PatientUrine36	human infant male urine patient 36
SAMN33342304	SRR23560174	PatientUrine37	human infant male urine patient 37
SAMN33342305	SRR23560173	PatientUrine38	human infant male urine patient 38
SAMN33342306	SRR23560172	PatientUrine39	human infant male urine patient 39
SAMN33342307	SRR23560171	PatientUrine40	human infant male urine patient 40
SAMN33342308	SRR23560169	PatientUrine41	human infant male urine patient 41
SAMN33342309	SRR23560168	PatientUrine42	human infant male urine patient 42
SAMN33342310	SRR23560167	PatientUrine43	human infant male urine patient 43
SAMN33342311	SRR23560166	PatientUrine44	human infant male urine patient 44
SAMN33342312	SRR23560165	PatientUrine45	human infant male urine patient 45
SAMN33342313	SRR23560164	PatientUrine46	human infant male urine patient 46
SAMN33342314	SRR23560163	PatientUrine47	human infant male urine patient 47
SAMN33342315	SRR23560162	PatientUrine48	human infant male urine patient 48
SAMN33342316	SRR23560161	PatientUrine49	human infant male urine patient 49
SAMN33342317	SRR23560160	PatientUrine50	human infant male urine patient 50
SAMN33342318	SRR23560158	NovaSeq_ExtractionBlank1	DNA extraction kit blank
SAMN33342319	SRR23560157	NovaSeq_ExtractionBlank2	DNA extraction kit blank
SAMN33342320	SRR23560156	NovaSeq_ExtractionBlank3	DNA extraction kit blank
SAMN33342321	SRR23560155	NovaSeq_ExtractionBlank4	DNA extraction kit blank
SAMN33342322	SRR23560154	NovaSeq_ExtractionBlank5	DNA extraction kit blank
SAMN33342323	SRR23560228	NovaSeq_ExtractionBlank6	DNA extraction kit blank
SAMN33342324	SRR23560227	NovaSeq_ExtractionBlank7	DNA extraction kit blank
SAMN33342325	SRR23560226	NovaSeq_ExtractionBlank8	DNA extraction kit blank
SAMN33342326	SRR23560225	NovaSeq_PCRBlank1	16S rRNA PCR amplification blank
SAMN33342327	SRR23560224	NovaSeq_PCRBlank2	16S rRNA PCR amplification blank
SAMN33342328	SRR23560222	NovaSeq_PCRBlank3	16S rRNA PCR amplification blank
SAMN33342329	SRR23560221	NovaSeq_PCRBlank4	16S rRNA PCR amplification blank
SAMN33342330	SRR23560220	NovaSeq_ZymoMockA	Zymo Mock Microbial community (undiluted)
SAMN33342331	SRR23560219	NovaSeq_ZymoMockB	Zymo Mock Microbial community (1/10 dilution)
SAMN33342332	SRR23560218	NovaSeq_ZymoMockC	Zymo Mock Microbial community (1/100 dilution)
SAMN33342333	SRR23560217	NovaSeq_ZymoMockD	Zymo Mock Microbial community (1/1000 dilution)
SAMN33342334	SRR23560216	NovaSeq_ZymoMockE	Zymo Mock Microbial community (10^-4 dilution)
SAMN33342335	SRR23560215	NovaSeq_ZymoMockF	Zymo Mock Microbial community (10^-5 dilution)
SAMN33342336	SRR23560214	NovaSeq_ZymoMockG	Zymo Mock Microbial community (10^-6 dilution)
SAMN33342337	SRR23560213	NovaSeq_ZymoMockH	Zymo Mock Microbial community (10^-7 dilution)
SAMN33342338	SRR23560211	SamplingControl1	Saline replicate 1
SAMN33342339	SRR23560210	SamplingControl2	Saline and catheter replicate 1
SAMN33342340	SRR23560209	SamplingControl3	mineral oil replicate 1
SAMN33342341	SRR23560208	SamplingControl4	Saline replicate 2
SAMN33342342	SRR23560207	SamplingControl5	saline and catheter replicate 2
SAMN33342343	SRR23560206	SamplingControl6	mineral oil replicate 2
SAMN33342344	SRR23560205	SamplingControl7	Saline replicate 3
SAMN33342345	SRR23560180	SamplingControl8	saline and catheter replicate 3
SAMN33342346	SRR23560179	SamplingControl9	mineral oil replicate 3
SAMN33342347	SRR23560178	SamplingControl10	saline replicate 4
SAMN33342348	SRR23560252	SamplingControl11	saline and catheter replicate 4
SAMN33342349	SRR23560251	SamplingControl12	mineral oil replicate 4

Supplementary_Methods–Infant Urobiome

Seth Reasoner Grace Morales Maria Hadjifrangiskou

2023-07-10

Contents

Introduction **1**

Processing of V4 16S rRNA reads using dada2 **1**
- Processing of 16S rRNA reads from Illumina MiSeq run . . . 1
- Processing of 16S rRNA reads from Illumina NovaSeq run . . . 3

Using Decontam to remove potential contaminant sequences **5**
- Merge Phyloseq Objects from Separate Sequencing Runs . . . 5
- Applying Decontam to mock communities to identify optimal Decontam threshold . . . 6
- Applying Decontam to remove potential contaminants within patient urine samples . . . 7

Figures **8**
- Figure 2A . . . 8
- Figure 2B . . . 8
- Figure 2C . . . 9
- Differential Abundance Testing . . . 9
- Figure 3 . . . 10

R session information **10**

Introduction

This file includes documentation detailing the code used for bioinformatics and statistical analysis accompanying the manuscript **Survey of the Infant Male Urobiome and Genomic Analysis of Actinotignum spp.**. Catheterized urine samples were collected from 50 healthy male infants. Following DNA extraction, the V4 hypervariable 16S rRNA region was amplified using the 515F and 806R primers. Sequences can be accessed under BioProject PRJNA912725. Supplementary Table S7 includes all sample accession information. Two sequencing runs were completed–one on a Illumina MiSeq and one on a Illumina NovaSeq. These runs and the samples sequenced are detailed in the manuscript text and Supplementary Table S1 & S7. Initial bioinformatic processing of 16S rRNA reads will be presented twice, once for each run. As detailed in Supplementary Table S7, **each sequencing run included 8 extraction blanks, 4 PCR negative controls, and 8 serial dilutions of a mock microbial community**.

Processing of V4 16S rRNA reads using dada2

Processing of 16S rRNA reads from Illumina MiSeq run

```
##Load requisite libraries (libraries used in each code chunk are re-called)
library("tidyverse")
```

```r
library("dada2")
library("here")
library("phyloseq")

##We would like to credit the following tutorial for guiding this code:
##"https://ycl6.github.io/16S-Demo/index.html"

##Create Folders
MiSeq_fastq = "/MiSeq_fastq/"        # raw fastq files
MiSeq_filtered = "/MiSeq_filtered"  # dada2 trimmed fastq files
MiSeq_output = "/MiSeq_output"      # output files
MiSeq_images = "/MiSeq_images"       # output images

if(!dir.exists(MiSeq_filtered)) dir.create(MiSeq_filtered)
if(!dir.exists(MiSeq_output)) dir.create(MiSeq_output)
if(!dir.exists(MiSeq_images)) dir.create(MiSeq_images)

##Create List of File Names
fns_MiSeq = sort(list.files(MiSeq_fastq, full.names = TRUE))
fnFwds_MiSeq = fns_MiSeq[grep("R1.fastq", fns_MiSeq)]
fnRevs_MiSeq = fns_MiSeq[grep("R2.fastq", fns_MiSeq)]

MiSeq_sample_names = gsub("_R1.fastq", "", basename(fnFwds_MiSeq))

##Visualize Read Quality
ii = 1:length(MiSeq_sample_names)
pdf(paste0(MiSeq_images, "/plotQualityProfile.pdf"), width = 8, height = 8, pointsize = 12)
for(i in ii) {
  message(paste0("[", i ,"/", length(MiSeq_sample_names), "] ", MiSeq_sample_names[i]))
  print(plotQualityProfile(fnFwds_MiSeq[i]) + ggtitle("Fwd"))
  print(plotQualityProfile(fnRevs_MiSeq[i]) + ggtitle("Rev"))
}
invisible(dev.off())

##Filter and Trim
filtFwds_MiSeq = file.path(MiSeq_filtered, basename(fnFwds_MiSeq))
filtRevs_MiSeq = file.path(MiSeq_filtered, basename(fnRevs_MiSeq))

out_MiSeq = filterAndTrim(fnFwds_MiSeq, filtFwds_MiSeq, fnRevs_MiSeq, filtRevs_MiSeq,
                    truncLen = c(140,140), minLen = 120, maxN = 0, truncQ = 2, maxEE =c(2,2),
                    rm.phix = TRUE, compress = TRUE, verbose = TRUE, multithread = TRUE)

out_MiSeq = as.data.frame(out_MiSeq)
rownames(out_MiSeq) = MiSeq_sample_names
head(out_MiSeq, 10)

##Learn the Error Rates
errFwd_MiSeq = learnErrors(filtFwds_MiSeq, multithread = TRUE)
errRev_MiSeq = learnErrors(filtRevs_MiSeq, multithread = TRUE)

pdf(paste0(MiSeq_images, "/plotErrors.pdf"), width = 10, height = 10, pointsize = 12)
plotErrors(errFwd_MiSeq, nominalQ = TRUE)
plotErrors(errRev_MiSeq, nominalQ = TRUE)
```

```
invisible(dev.off())

##Sample Inference
dadaFwds_MiSeq = dada(filtFwds_MiSeq, err = errFwd_MiSeq, pool = FALSE, multithread = TRUE)
dadaRevs_MiSeq = dada(filtRevs_MiSeq, err = errRev_MiSeq, pool = FALSE, multithread = TRUE)

##Merge Paired Reads
merged_MiSeq = mergePairs(dadaFwds_MiSeq, filtFwds_MiSeq, dadaRevs_MiSeq, filtRevs_MiSeq, verbose = TRU

##Construct the Sequence Table
seqtab_MiSeq <- makeSequenceTable(merged_MiSeq)

table(nchar(getSequences(seqtab_MiSeq)))

##Remove Chimeras
seqtab_nochim_MiSeq <- removeBimeraDenovo(seqtab_MiSeq, method="consensus", multithread=2, verbose=TRUE
##Percent of sequences that are non-chimeric
sum(seqtab_nochim_MiSeq)/sum(seqtab_MiSeq)

rownames(seqtab_nochim_MiSeq) <- MiSeq_sample_names

##Track Reads through the Pipeline
getN <- function(x) sum(getUniques(x))
track_MiSeq <- cbind(out_MiSeq, sapply(dadaFwds_MiSeq, getN), sapply(dadaRevs_MiSeq, getN), sapply(merge
colnames(track_MiSeq) <- c("input", "filtered", "denoisedF", "denoisedR", "merged","nonchim")
rownames(track_MiSeq) <- MiSeq_sample_names
head(track_MiSeq)

##Assign Taxonomy
seqs_MiSeq = getSequences(seqtab_nochim_MiSeq)
dbpath = "/16S_DB/"
ref_db = paste0(dbpath, "silva_nr99_v138.1_train_set.fa.gz")

taxonomy_tab_MiSeq <- assignTaxonomy(seqs_MiSeq, refFasta = ref_db)


##Create Phyloseq Object
MiSeq_phyloseq_unfiltered <- phyloseq(otu_table(seqtab_nochim_MiSeq, taxa_are_rows=FALSE),
               tax_table(taxonomy_tab_MiSeq))
```

Processing of 16S rRNA reads from Illumina NovaSeq run

```
##Load requisite libraries
library("tidyverse")
library("dada2")
library("here")
library("phyloseq")

##Create Folders
Nova_fastq = "/Nova_fastq/"        # raw fastq files
Nova_filtered = "/Nova_filtered"  # dada2 trimmed fastq files
Nova_output = "/Nova_output"      # output files
Nova_images = "/Nova_images"       # output images
```

```r
if(!dir.exists(Nova_filtered)) dir.create(Nova_filtered)
if(!dir.exists(Nova_output)) dir.create(Nova_output)
if(!dir.exists(Nova_images)) dir.create(Nova_images)

##Create List of File Names
fns_Nova = sort(list.files(Nova_fastq, full.names = TRUE))
fnFwds_Nova = fns_Nova[grep("R1.fastq", fns_Nova)]
fnRevs_Nova = fns_Nova[grep("R2.fastq", fns_Nova)]

Nova_sample_names = gsub("_R1.fastq", "", basename(fnFwds_Nova))

##Visualize Read Quality
ii = 1:length(Nova_sample_names)
pdf(paste0(Nova_images, "/plotQualityProfile.pdf"), width = 8, height = 8, pointsize = 12)
for(i in ii) {
  message(paste0("[", i ,"/", length(Nova_sample_names), "] ", Nova_sample_names[i]))
  print(plotQualityProfile(fnFwds_Nova[i]) + ggtitle("Fwd"))
  print(plotQualityProfile(fnRevs_Nova[i]) + ggtitle("Rev"))
}
invisible(dev.off())

##Filter and Trim
filtFwds_Nova = file.path(Nova_filtered, basename(fnFwds_Nova))
filtRevs_Nova = file.path(Nova_filtered, basename(fnRevs_Nova))

out_Nova = filterAndTrim(fnFwds_Nova, filtFwds_Nova, fnRevs_Nova, filtRevs_Nova,
                    truncLen = c(140,140), minLen = 120, maxN = 0, truncQ = 2, maxEE =c(2,2),
                    rm.phix = TRUE, compress = TRUE, verbose = TRUE, multithread = TRUE)

out_Nova = as.data.frame(out_Nova)
rownames(out_Nova) = Nova_sample_names
head(out_Nova, 10)

##Learn the Error Rates
errFwd_Nova = learnErrors(filtFwds_Nova, multithread = TRUE)
errRev_Nova = learnErrors(filtRevs_Nova, multithread = TRUE)

pdf(paste0(Nova_images, "/plotErrors.pdf"), width = 10, height = 10, pointsize = 12)
plotErrors(errFwd_Nova, nominalQ = TRUE)
plotErrors(errRev_Nova, nominalQ = TRUE)
invisible(dev.off())

##Sample Inference
dadaFwds_Nova = dada(filtFwds_Nova, err = errFwd_Nova, pool = FALSE, multithread = TRUE)
dadaRevs_Nova = dada(filtRevs_Nova, err = errRev_Nova, pool = FALSE, multithread = TRUE)

##Merge Paired Reads
merged_Nova = mergePairs(dadaFwds_Nova, filtFwds_Nova, dadaRevs_Nova, filtRevs_Nova, verbose = TRUE)

##Construct the Sequence Table
seqtab_Nova <- makeSequenceTable(merged_Nova)

table(nchar(getSequences(seqtab_Nova)))
```

```
##Remove Chimeras
seqtab_nochim_Nova <- removeBimeraDenovo(seqtab_Nova, method="consensus", multithread=2, verbose=TRUE)
##Percent of sequences that are non-chimeric
sum(seqtab_nochim_Nova)/sum(seqtab_Nova)

rownames(seqtab_nochim_Nova) <- Nova_sample_names

##Track Reads through the Pipeline
getN <- function(x) sum(getUniques(x))
track_Nova <- cbind(out_Nova, sapply(dadaFwds_Nova, getN), sapply(dadaRevs_Nova, getN), sapply(merged_N
colnames(track_Nova) <- c("input", "filtered", "denoisedF", "denoisedR", "merged","nonchim")
rownames(track_Nova) <- Nova_sample_names
head(track_Nova)

##Assign Taxonomy
seqs_Nova = getSequences(seqtab_nochim_Nova)
dbpath = "/16S_DB/"
ref_db = paste0(dbpath, "silva_nr99_v138.1_train_set.fa.gz")

taxonomy_tab_Nova <- assignTaxonomy(seqs_Nova, refFasta = ref_db)

##Create Phyloseq Object
Nova_phyloseq_unfiltered <- phyloseq(otu_table(seqtab_nochim_Nova, taxa_are_rows=FALSE),
               tax_table(taxonomy_tab_Nova))
```

Using Decontam to remove potential contaminant sequences

We use the R package Decontam (citation: PMID 30558668) to identify sequences that are more prevalent in the negative controls (sampling controls, extraction blanks and PCR blanks) than the patient samples. First, using the known composition of the mock microbial community from each sequencing run, we benchmarked Decontam performance to retain true sequence variants. Next, we applied the same threshold to patient samples to remove contaminants.

Merge Phyloseq Objects from Separate Sequencing Runs

```
##Load requisite libraries
library("tidyverse")
library("phyloseq")

merged_output <- merge_phyloseq(MiSeq_phyloseq_unfiltered, Nova_phyloseq_unfiltered)

# add metadata
complete_metadata<- read.csv("https://raw.githubusercontent.com/reaset41/Infant-Urobiome/main/metadata_

sample_data(merged_output) <- complete_metadata

# this merged phyloseq object is available on the associated github
urobiome_phyloseq <- merged_output

# export ASV table prior to decontamination (Table S2)
full_tax <- tax_table(urobiome_phyloseq)
full_ASV <- otu_table(urobiome_phyloseq)
```

```
TableS2 <- cbind(full_tax,t(full_ASV))

write.csv(TableS2, "TableS2.csv", row.names=TRUE)
```

Applying Decontam to mock communities to identify optimal Decontam threshold

```
##Load requisite libraries
library("tidyverse")
library("decontam")
library("phyloseq")

##We would like to credit the following publication for guiding this code: Controlling for Contaminants
##https://lakarstens.github.io/ControllingContaminants16S/Analyses/ControllingContaminants16S_decontam.

##Using merged phyloseq object created above
urobiome_phyloseq

##Create subset with only mock communities
mock_phyloseq <-subset_samples(urobiome_phyloseq, SampleType=="Mock")
#mock community includes 8 bacteria
sorted <- head(sort(colSums(otu_table(mock_phyloseq)), decreasing = TRUE), 8)
mock_taxa <- cbind(as.data.frame(tax_table(mock_phyloseq)[names(sorted),]), Count = sorted)

##Create subset of data with only mock and negative controls
mock_blanks <- subset_samples(urobiome_phyloseq, !SampleType=="PATIENT")

##extract out original ASV table
original_ASVs <- as.matrix(as.data.frame(otu_table(mock_blanks)))

##identify the original proportion of contaminants
contaminants_original <- rowSums(original_ASVs[,!colnames(original_ASVs) %in% rownames(mock_taxa)])
##identify the original proportion of mock community ASVs
mock_original <- rowSums(original_ASVs[,colnames(original_ASVs) %in% rownames(mock_taxa)])

##Evaluate different thresholds of Decontam
contam_Mocks <- isNotContaminant(mock_blanks, neg = "is.neg", method = "prevalence", threshold = 0.4, n

##create a phyloseq object without contaminants
mock_blank_true <- prune_taxa(contam_Mocks$not.contaminant, mock_blanks)

##remove ASVs <1% prevalence in entire dataset
filter_level_mocks <- colSums(otu_table(mock_blank_true))>(0.01*sum(rowSums(otu_table(mock_blank_true))
mocks_final <-prune_taxa(filter_level_mocks, mock_blank_true)

##subset out the ASV table of recovered ASVs
recovered_ASVs <- as.matrix(as.data.frame(otu_table(mock_blank_true)))

##create a subset of removed ASVs
removed_ASVs <- as.matrix(as.data.frame(otu_table(mock_blanks)))
removed_ASVs <- removed_ASVs[,!colnames(removed_ASVs) %in% colnames(recovered_ASVs)]

# Summarize results
##proportion of contaminants removed (of all total contaminant ASVs)
```

```
contaminants_removed = (rowSums(removed_ASVs[,!colnames(removed_ASVs) %in% rownames(mock_taxa)])/ contam
##proportion of mock removed (of all total mock ASVs)
mock_ASVs_removed = removed_ASVs[,(colnames(removed_ASVs) %in% rownames(mock_taxa))]/ mock_original

##total amount of contaminants remaining in new phyloseq object
contaminants_remaining =  rowSums(recovered_ASVs[,!colnames(recovered_ASVs) %in% rownames(mock_taxa)])

# Calculate classifications

##Percent of mock community ASVs correctly classified as mock community ASVs
true_neg <- rowSums(recovered_ASVs[,colnames(recovered_ASVs) %in% rownames(mock_taxa)])
##Percent of mock community incorrectly classified as mock community ASVs
false_neg <- rowSums(recovered_ASVs[,!colnames(recovered_ASVs) %in% rownames(mock_taxa)])
##identify non-mock community ASVs correctly classified as not belonging to mock community
true_pos <- rowSums(removed_ASVs[,!colnames(removed_ASVs) %in% rownames(mock_taxa)])
# #identify mock community ASVs incorrectly classified as not belonging to mock community
false_pos <- rowSums(removed_ASVs[,colnames(removed_ASVs) %in% rownames(mock_taxa)])

sensitivity <- true_pos/(true_pos + false_neg)
specificty <- true_neg/(true_neg + false_pos)
accuracy <- (true_pos + true_neg) / (false_pos + true_pos + false_neg + true_neg)
prevalence <- (true_pos + false_neg) / (false_pos + true_pos + false_neg + true_neg)

##Following testing various thresholds, we settled on 0.4 as the ideal threshold for identifying contam
```

Applying Decontam to remove potential contaminants within patient urine samples

```
##Load requisite libraries
library("tidyverse")
library("decontam")
library("phyloseq")

##Using phyloseq object created above
urobiome_phyloseq

##create subset of data excluding mock community samples
Patient_Blanks <- subset_samples(urobiome_phyloseq, !SampleType =="Mock")

##apply Decontam
contam_Patient <- isNotContaminant(Patient_Blanks, neg = "is.neg", method ="prevalence",threshold = 0.4

##create physloseq object without contaminants
patient_true_blanks <- prune_taxa(contam_Patient$not.contaminant, Patient_Blanks)

##remove ASVs <1% prevalence in entire dataset
filter_level <- colSums(otu_table(patient_true_blanks))>(0.01*sum(rowSums(otu_table(patient_true_blanks
patient_true <- prune_taxa(filter_level, patient_true_blanks)

##Subset to patient results only
patient_true <- subset_samples(patient_true, SampleType=="PATIENT")

##remove chloroplast, mitochondrial, and unspecified taxa
```

```
patient_true_final <-subset_taxa(patient_true, Kingdom == "Bacteria"& Family != "Mitochondria" & Genus

## this is Table S3
patient_tax <- tax_table(patient_true_final)
patient_ASV <- otu_table(patient_true_final)
TableS3 <- cbind(patient_tax,t(patient_ASV))

write.csv(TableS3, "TableS3.csv", row.names=TRUE)
```

Figures

Figure 2A

```
##Load requisite libraries
library(tidyverse)
library(phyloseq)
library(vegan)

# beta diversity prior to decontamination

patient_sampling <- subset_samples(urobiome_phyloseq, SampleType == c("PATIENT","SamplingControl"))
GroupsColors <- c("#0072b2","#D81B60")

dist <- phyloseq::distance(patient_sampling, method = "bray")
ord <- ordinate(patient_sampling, method="PCoA", distance = "bray")

Figure2A <- plot_ordination(patient_sampling, ord, color = "SampleType") +
  geom_point(size=3) +
  stat_ellipse(aes(group=SampleType))+
  scale_color_manual(values = GroupsColors)+
  theme_bw()+
  theme(panel.grid = element_blank(),
        panel.border = element_rect(color="black",fill=NA,size=1.5),
        axis.text=element_text(size=13),
        axis.title=element_text(size=18))
        panel.background = element_rect(fill = "transparent"),
        plot.background = element_rect(fill = "transparent", color = NA),
        legend.background = element_rect(fill = "transparent"))


samples <- data.frame(sample_data(patient_sampling))
adonis2(dist ~ Figure2A, data = samples)
```

Figure 2B

```
##Load requisite libraries
library("tidyverse")
library("microbiome")
library("phyloseq")

sample_list <- c("H1", "H2", "H3", "H4", "H5", "H6", "H7", "H8", "H9", "H10", "H11", "H12", "H13", "H14
```

```
Figure2B <- comp_barplot(patient_true_final, tax_level = "Genus", n_taxa = 6, bar_width = 0.7, sample_o
  theme(axis.text.x = element_text(size=12),
        axis.text.y = element_text(size=7),
        axis.title.x = element_text(size=15))
```

Figure 2C

```
##Load requisite libraries
library("tidyverse")
library("decontam")
library("phyloseq")
library("ggpubr")

alpha_stats <- estimate_richness(patient_true_final, measures = c("Chao1", "Shannon"))
GroupsColors <- c("#6db6ff", "#009292")
comparisons_delivery <- list( c("Vaginal", "Caesarean"))
comparisons_abx <- list( c("Yes", "No"))
symnum.args = list(cutpoints = c(0, 0.0001, 0.001, 0.01, 0.05, 1), symbols = c("****", "***", "**", "*"

alpha_stats <- estimate_richness(patient_true_final, measures = c("Chao1", "Shannon"))
GroupsColors <- c("#6db6ff", "#009292")
comparisons_delivery <- list( c("Vaginal", "Caesarean"))
comparisons_abx <- list( c("Yes", "No"))
symnum.args = list(cutpoints = c(0, 0.0001, 0.001, 0.01, 0.05, 1), symbols = c("****", "***", "**", "*"

Delivery_Stat <- plot_richness(patient_true_final, x="Delivery_Mode", measures=c("Chao1", "Shannon"), c
  geom_boxplot(alpha=0.6, size=1.5)+
  geom_point(size=4)+
  theme_bw()+
  theme(axis.text = element_text(size = 16))+
  scale_color_manual(values = GroupsColors)+
  theme(legend.position="none", axis.text.x=element_text(angle=0,hjust=0.5,vjust=1,size=8))+
  stat_compare_means(method = "wilcox.test", comparisons = comparisons_delivery, label = "p.signif", sy

Abx_Stat <- plot_richness(patient_true_final, x="PriorAbx", measures=c("Chao1", "Shannon"), color = "Pr
  geom_boxplot(alpha=0.6, size=1.5)+
  geom_point(size=4)+
  theme_bw()+
  theme(axis.text = element_text(size = 16))+
  scale_color_manual(values = GroupsColors)+
  theme(legend.position="none", axis.text.x=element_text(angle=0,hjust=0.5,vjust=1,size=9))+
  stat_compare_means(method = "wilcox.test", comparisons = comparisons_abx, label = "p.signif", symnum.a
```

Differential Abundance Testing

```
##Load requisite libraries
library("tidyverse")
library("phyloseq")
library("Maaslin2")

ASV_input_data <- read.csv("https://raw.githubusercontent.com/reaset41/Infant-Urobiome/main/asvs_maasli
metadata_maaslin2_urobiome <-read.csv("https://raw.githubusercontent.com/reaset41/Infant-Urobiome/main/
```

```r
urobiome_maaslin2 <- Maaslin2(
  input_data = ASV_input_data,
  input_metadata = metadata_maaslin2_urobiome,
  min_prevalence = 0.1,
  min_abundance = 0.0,
  normalization = "TSS",
  transform= "NONE",
  output = "test_output_ASVs_LM",
  fixed_effects = c("DeliveryMode","PriorAbx","Age_days"),
  reference = c("DeliveryMode,Vaginal","PriorAbx,No"),
  analysis_method='LM',
  correction='BH',
  max_significance = 0.25,
  plot_heatmap = FALSE,
  plot_scatter = FALSE)
```

Figure 3

```r
library(tidyverse)
library(reshape2)

concordance <- read.csv("https://raw.githubusercontent.com/reaset41/Infant-Urobiome/main/concordance_re

melt_concord <- melt(concordance)

##0 = not detected
##1 = EQUC only
##2 = 16S rRNA only
##3 = Both methods
col1 <- c("0", "1", "2", "3")

Concord <- ggplot(melt_concord, aes(variable, Family, fill=factor(value))) +
  geom_tile(color = "white",linewidth = 0.25,linetype = 1)+
  coord_fixed()+
  scale_fill_manual(values = c("grey", "#800000", "#ADD8E6", "#000080"))+
  xlab("Patient Number")+
  theme(
    axis.text.x   = element_text(colour = "black", angle=45, size=5),
    axis.title.x = element_text(colour = "black", size = rel(0.5)),
    axis.text.y = element_text(colour = "black", size=5))
```

R session information

```r
sessionInfo()
```

```
## R version 4.2.1 (2022-06-23)
## Platform: x86_64-apple-darwin17.0 (64-bit)
##
## Matrix products: default
## BLAS:   /Library/Frameworks/R.framework/Versions/4.2/Resources/lib/libRblas.0.dylib
## LAPACK: /Library/Frameworks/R.framework/Versions/4.2/Resources/lib/libRlapack.dylib
```

```
##
## locale:
## [1] en_US.UTF-8/en_US.UTF-8/en_US.UTF-8/C/en_US.UTF-8/en_US.UTF-8
##
## attached base packages:
## [1] stats     graphics  grDevices utils     datasets  methods   base
##
## loaded via a namespace (and not attached):
##  [1] digest_0.6.31   lifecycle_1.0.3 magrittr_2.0.3  evaluate_0.19
##  [5] rlang_1.0.6     stringi_1.7.8   cli_3.5.0       rstudioapi_0.14
##  [9] vctrs_0.5.1     rmarkdown_2.19  tools_4.2.1     stringr_1.5.0
## [13] glue_1.6.2      xfun_0.36       yaml_2.3.6      fastmap_1.1.0
## [17] compiler_4.2.1  htmltools_0.5.4 knitr_1.41
```

Description of Additional Supplementary Files

File Name: Supplementary Data 1
Description: Amplicon sequence variants (ASVs) in all samples (urine samples, negative controls, sampling controls). ASVs prior to decontamination. ASVs were inferred using the DADA2 pipeline.

File Name: Supplementary Data 2
Description: ASVs in subject urine samples (n=50) retained following removal of contaminating ASVs. Contaminating sequences were removed using the R package Decontam.

File Name: Supplementary Methods
Description: This file contains the PDF exported version of an RMarkdown document. This document contains all bioinformatic code used for processing of raw sequencing data, decontamination of sequencing data, and plotting figures.
